# Chemical Exposures, DNA Methylation and Whole Blood DNA Damage in Pet Golden Retrievers

**DOI:** 10.1111/vco.70066

**Published:** 2026-04-19

**Authors:** Ashleigh N. Tindle, Mark E. Berres, Lauren Baker, Zhuonan Wu, Brenna Swafford, Julia Labadie, Lauren A. Trepanier

**Affiliations:** ^1^ Department of Medical Sciences University of Wisconsin‐Madison Madison Wisconsin USA; ^2^ Bioinformatics Resource Center University of Wisconsin‐Madison Madison Wisconsin USA; ^3^ Department of Animal and Dairy Sciences University of Wisconsin‐Madison Madison Wisconsin USA; ^4^ Scientific Programs Department Morris Animal Foundation Denver Colorado USA

## Abstract

Canine multicentric lymphoma (CL) is a common and typically fatal cancer among dogs. Although certain breeds have a higher incidence of CL, its environmental risk factors remain uncertain. Exposures to herbicides and volatile organic compounds (VOCs) associate with non‐Hodgkin lymphoma in people. These exposures also correlate with measurable in vivo DNA strand breaks in dogs, even at estimated systemic concentrations that do not reach genotoxic thresholds. Herbicides and VOCs can also exert genotoxicity through differential DNA methylation. We therefore hypothesised that higher chemical exposures would be associated with differential global and gene‐specific methylation in the blood of pet golden retrievers, and that differential gene‐specific methylation would be further associated with measured DNA strand breaks and oxidised DNA damage. We performed Oxford Nanopore sequencing in 24 of 60 dogs from a recent case–control study within the Golden Retriever Lifetime Study cohort, selected based on the highest and lowest quintiles of estimated aggregate herbicide and VOC exposures. Contrary to our initial hypothesis, higher estimated chemical exposures were associated with relatively lower promoter methylation of antioxidant genes (median 0.335 versus 0.374; *p* = 0.007) and 8‐oxoguanine DNA repair genes (median 0.254 versus 0.294; *p* = 0.007). This pattern could represent a compensatory response to chemical exposures rather than a driver of DNA damage. Follow‐up studies are needed to determine whether these modest promoter methylation differences correspond to biologically meaningful increases in gene expression, antioxidant status and DNA repair capacity.

## 1 Introduction

Canine multicentric lymphoma (CL) is one of the most common cancers in pet dogs, with many common molecular and clinical characteristics to human non‐Hodgkin lymphoma (NHL) [1]. The incidence of CL, like NHL, is increased in industrial regions [2, 3, 4], and even tracks to similar geographic regions with human NHL [5, 6], which suggests shared environmental risk factors.

Both NHL and CL are associated with exposures to herbicides and volatile organic compounds (VOCs) [7, 8, 9, 10, 11]. In addition, the herbicides 2,4‐D and glyphosate and the VOCs benzene and xylene are genotoxic to canine lymphoid cells at micromolar concentrations [12, 13]. Further, in vivo exposures to these chemicals are ubiquitous in pet dogs, with some dogs reaching estimated blood concentrations that reached in vitro genotoxic thresholds [12, 13].

Estimated systemic herbicide and VOC exposures were positively correlated with DNA strand breaks in the whole blood of pet golden retrievers [14], a breed at increased risk for CL [15]. In addition, oxidised DNA damage was higher in the year before CL diagnosis compared to unaffected controls at the same time point [14].

Herbicides and VOCs can directly cause DNA strand breaks in canine cells in vitro [12, 13], and can generate DNA damage through oxidative stress [16, 17]. However, these chemicals can also exert genotoxicity at even lower concentrations through epigenetic mechanisms. For example, global DNA hypomethylation leads to genomic instability [18], and glyphosate and benzene induce global DNA hypomethylation in human lymphoid cells in vitro at low micromolar concentrations [19, 20]. Herbicides and VOCs can also cause differential methylation of specific genes, including those involved in DNA repair [21, 22]. Based on these findings, we hypothesised that higher chemical exposures would be associated with differential global and gene‐specific methylation in the blood of pet golden retrievers, and that differential gene‐specific promoter methylation would be further associated with measured DNA strand breaks and oxidised DNA damage.

The objectives of this study were to (1) determine whether estimated aggregate herbicide and VOC exposures were associated with global DNA hypomethylation in golden retriever dogs; (2) assess whether aggregate herbicide and VOC exposures were associated with differential promoter methylation of genes involved in redox regulation or DNA repair in peripheral blood and (3) explore whether methylation patterns in these genes were associated with observed DNA strand breaks or oxidised DNA residues. Our specific hypotheses were that higher chemical exposures would be associated with global DNA hypomethylation (a marker of genomic instability), targeted promoter hypermethylation (silencing) of genes involved in antioxidant and DNA repair pathways, and promoter hypomethylation (activation) of pro‐oxidant genes. We further hypothesised that promoter hypermethylation of genes involved in antioxidant and DNA repair pathways, and promoter hypomethylation of pro‐oxidant genes, would associate with higher measured DNA strand breaks and oxidised DNA residues.

## 2 Methods

### 2.1 Dog Demographics

Dogs for the current study were a subgroup from a nested case–control study population of 30 golden retrievers with CL and 30 matched unaffected controls [12, 13] that originated from the Golden Retriever Lifetime Study (GRLS) longitudinal cohort [23]. The current study population included 24 dogs with the highest (*n* = 12) and lowest (*n* = 12) quintiles of estimated aggregate herbicide and VOC exposures [12, 13]. All dogs had banked urine and whole blood in EDTA made available by the GRLS in the year prior to CL diagnosis or comparable time point for controls.

### 2.2 Chemical Exposures, DNA Strand Breaks and Oxidised DNA Residues

**FIGURE 1:**
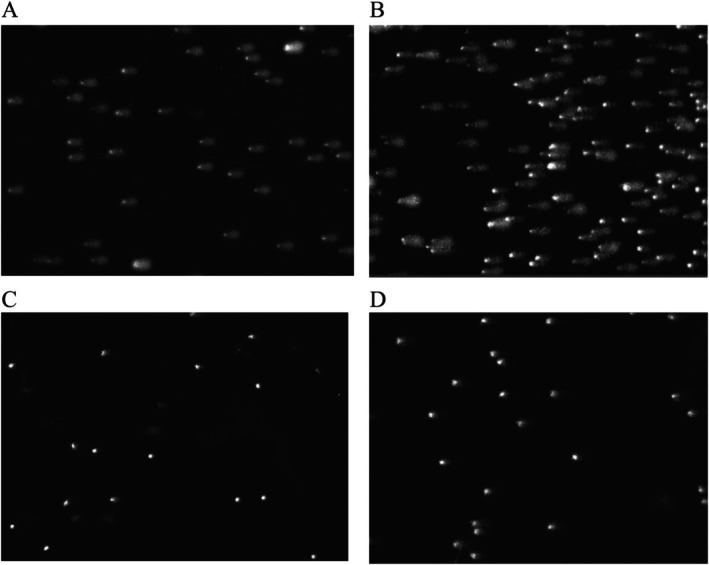
Representative comet assays for DNA damage in the peripheral blood of a golden retriever in the highest quintile of herbicide and VOC exposures, in the year prior to diagnosis of multicentric lymphoma (A and B) and a golden retriever in the lowest quintile of herbicide and VOC exposures, who was unaffected by lymphoma in the subsequent year (C and D) [14]. Panels to the right represent assays run with the addition of the enzyme FPG, which recognizes oxidized DNA residues and converts them into DNA strand breaks that can be detected by the comet assay [24]. Comet tails, which denote DNA strand breaks, were quantified as % DNA in the comet tail with Comet Assay Analysis software (CAS).

Aggregate systemic herbicide and VOC exposures were estimated using reverse dosimetry from urinary herbicide and VOC metabolite concentrations [14]. DNA strand breaks in banked whole blood were measured using the alkaline comet assay. Oxidised DNA residues were assessed using the comet assay with the addition of formamidopyrimidine DNA glycosylase (FPG), an enzyme that recognises oxidised DNA residues (particularly 8‐oxoguanine) and converts them into DNA strand breaks detectable by the comet assay [14]. A representative comet assay is shown in Figure 1, from two golden retrievers in the highest and lowest quintiles of estimated chemical exposures [12, 13, 14].

### 2.3 Oxford Nanopore Sequencing

The Oxford Nanopore long‐read sequencing platform was used to assess global and gene‐specific methylation. The Oxford Nanopore platform performs comparably to bisulfite sequencing in detecting DNA methylation [25] and additionally provides sequence information (single nucleotide polymorphisms, indels and translocations) to support comprehensive downstream hypothesis testing in this unique cohort.

Banked whole blood samples were provided by the GRLS and served as the starting material for DNA extraction and library preparation prior to long‐read sequencing. Selected samples were submitted to the University of Wisconsin‐Madison Biotechnology Center for DNA library preparation and sequencing. High molecular weight (HMW) genomic DNA was isolated using Nanobind CBB Big DNA Kit (PacBio) following the manufacturer's HMW DNA Extraction Protocol. DNA purity and concentration were measured on a NanoDrop One instrument (Thermo Fisher Scientific) and DNA concentration was verified using the Qubit dsDNA HS Assay Kit (Thermo Fisher Scientific). DNA size, integrity and quality were assessed on the Femto Pulse System (Agilent Technologies).

Purified DNA was sheared to approximately15–20 kb with a Covaris G Tube (Perkin Elmer). Libraries were prepared from the sheared DNA using the SQK‐LSK114 kit (Oxford Nanopore Technologies) following the manufacturer's recommendations. Library concentrations were quantified using the Qubit dsDNA High Sensitivity kit (ThermoFisher Scientific). Sequencing was performed on FLO‐PRO114M (R10.4.1) flow cells using the PromethION 24 Nanopore sequencer. Flow cells were flushed between sequence reads with the ONT Flow Cell Wash Kit (EXP‐WSH004) and reloaded with fresh library according to the manufacturer's instructions.

Oxford Nanopore LSK‐114 sequencing runs were conducted for 72 h, generating approximately 8.62 million reads and yielding about 89 Gb of data per sample, with a read N50 of 12.5 Kb. Base calling was performed using Dorado (v0.7.3‐linux‐x64) with the super‐accuracy (SUP) model dna_r10.4.1_e8.2_400bps_sup@v4.2.0. DNA methylation calling was performed with modkit (v0.3.1) using the options ‐‐cpg ‐‐combine‐strands and, aligned to the canine reference genome (Dog10K_Boxer_Tasha, GCF_000002285.5). Per‐CpG methylation frequencies, along with counts of methylated and total reads and genomic coordinates, were summarised in BED‐format files for downstream analysis. For all methylation calls, corrected files were used in which C‐to‐T single nucleotide variants (SNVs) were filtered based on sample‐specific variant calls determined by clair3, version 1.0.9 [26]. These corrections reduce false‐positive methylation calls and ensure higher‐confidence base modification measurements.

### 2.4 Global Analyses

Global DNA methylation was assessed using SNV‐corrected bedMethyl files generated from Oxford Nanopore sequencing data. CpG sites were reported as either modified (5mCpG; methylated) or unmodified CpG (unmethylated); global methylation was calculated as methylated CpG calls divided by total CpG calls (5mCpG plus unmodified CpG) across the genome.

### 2.5 Gene‐Specific Promoter Methylation

We assessed promoter methylation in 67 genes identified a priori that could be relevant to in vivo DNA damage, with some overlap in function for some genes: 38 genes involved in the repair of DNA strand breaks, 5 genes involved in repair of 8‐oxoguanine residues and 32 genes related to systemic redox stress (listed in Tables S1 and S2). Promoter regions were defined as −1000 bp upstream from the transcription start site [27]. Promoter regions were identified by using the start of each gene transcript in custom BED files and then filtered and applied to gene coordinates extracted from the Dog10K Boxer (Tasha) reference genome Gene Transfer Format file. CpG methylation calls from Oxford Nanopore sequencing were then intersected with promoter regions using bedtools v2.29.2 with command bedtools intersect, −wa and −wb, linking each CpG site to a specific gene promoter [25, 28]. For each gene, mean promoter methylation was calculated by dividing the total number of methylated reads by the sum of methylated and unmethylated reads across all CpG sites within the designated promoter region. CpG sites with low coverage (< 10 total reads) were excluded prior to summarisation [26].

### 2.6 Statistical Methods

The genome‐wide proportion of unmethylated CpG sites was compared between high and low quintile chemical exposure groups using an unpaired *t*‐test. Mean methylation values across CpG sites within the promoters of selected genes were further averaged within the gene groups involved in DNA strand break repair, 8‐oxoguanine repair, antioxidant pathways, or pro‐oxidant pathways. These promoter methylation values by gene group were then compared between high and low quintiles of aggregate chemical exposures using unpaired *t‐*tests or Mann–Whitney *U* tests, depending on data distribution. Data are shown as medians and observed ranges across all groups for consistency. Multivariable linear regression was used to evaluate any positive associations between exposure and mean promoter methylation for interactions with age, sex/neuter status, or case/control status.

Associations between individual chemical exposures (specifically 2,4‐d, glyphosate and xylene) [14] and promoter methylation for the selected groups of genes were tested with simple linear regression. Multivariable linear regression was used to model any positive associations between individual exposures and mean promoter methylation for interactions with age, sex/neuter and case/control status.

Simple linear regression was also used to test associations between measured DNA strand breaks or oxidised DNA residues and promoter methylation across relevant gene groups (DNA strand break or 8‐oxoguanine repair, respectively; antioxidant pathways, or pro‐oxidant pathways). All statistical analyses were completed in RStudio (posit.co) with *p* < 0.05 considered significant.

## 3 Results

### 3.1 Dog Demographics

A total of 24 dogs were included in the analysis, with 12 dogs in each of the highest and lowest quintiles of aggregate chemical exposures (Table 1) from previous case–control studies [12, 13]. The highest exposure group had a median age of 6.2 years and included eight CL cases; the lowest exposure group had a median age of 5.8 years and included five CL cases.

**TABLE 1:** Pet golden retrievers grouped by estimated aggregate systemic exposures to genotoxic chemicals (the herbicides 2,4‐D and glyphosate and the volatile organic compounds (VOCs) benzene, xylene and 1,3‐butadiene) from two previous case–control studies [12, 13].

	Highest exposure quintile	Lowest exposure quintile
Number of dogs	12	12
Median age (observed range)	6.2 years (1.8–9.4)	5.8 years (2.6–9.9)
Sex/neuter status (*n*)	FS 5 FI 1 MN 6 MI 0	FS 2 FI 1 MN 6 MI 3
Case/control status	Lymphoma 8 Healthy control 4	Lymphoma 5 Healthy control 7
Range of estimated aggregate systemic chemical exposures	5.60–26.48 μM	1.45–2.93 μM

*Note:* Peripheral blood was assayed for DNA strand breaks and oxidised DNA residues, and was then subjected to methylomics analyses using Oxford Nanopore sequencing. Dogs in each exposure group contained a mix of lymphoma cases and unaffected controls.

### 3.2 Global DNA Hypomethylation

**FIGURE 2:**
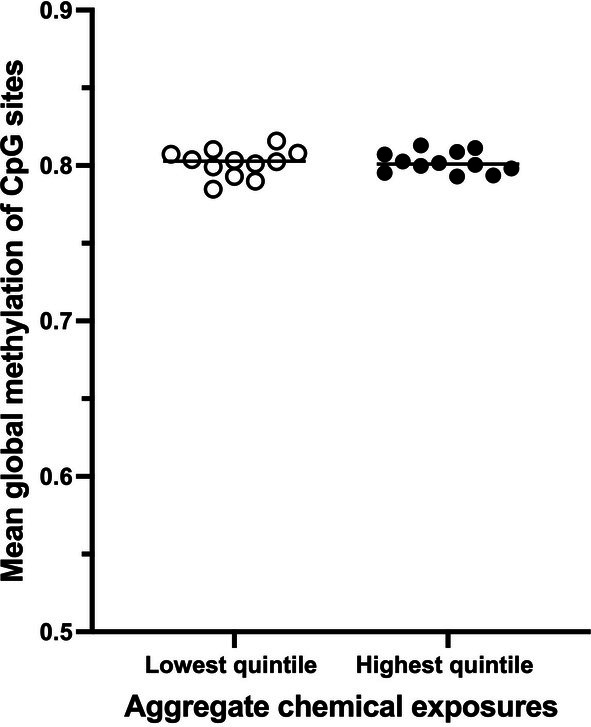
Global DNA hypomethylation, represented by unmodified CpG sites detected by Oxford Nanopore sequencing, in the whole blood of 24 pet golden retriever dogs. Dogs are grouped by the highest and lowest quintiles of estimated aggregate systemic exposures for the herbicides 2,4‐D and glyphosate and the volatile organic compounds benzene, xylene and 1,3‐butadiene out of 60 dogs screened [12, 13]. Horizontal line represents both the median and the mean for each group. *p* = 0.88 between exposure groups.

We did not find evidence of global DNA hypomethylation with higher aggregate herbicide and VOC exposures: the proportions of methylated CpG sites across the genome were comparable between dogs with the highest and lowest quintiles of chemical exposure (median 0.80, observed range 0.79–0.81; and median 0.80, observed range 0.78–0.82, respectively; *p* = 0.88, Figure 2). A genomic map of methylation sites by chromosome for high and low chemical exposure groups is provided in Figure S1.

### 3.3 Aggregate Chemical Exposures and DNA Damage Pathways

**FIGURE 3:**
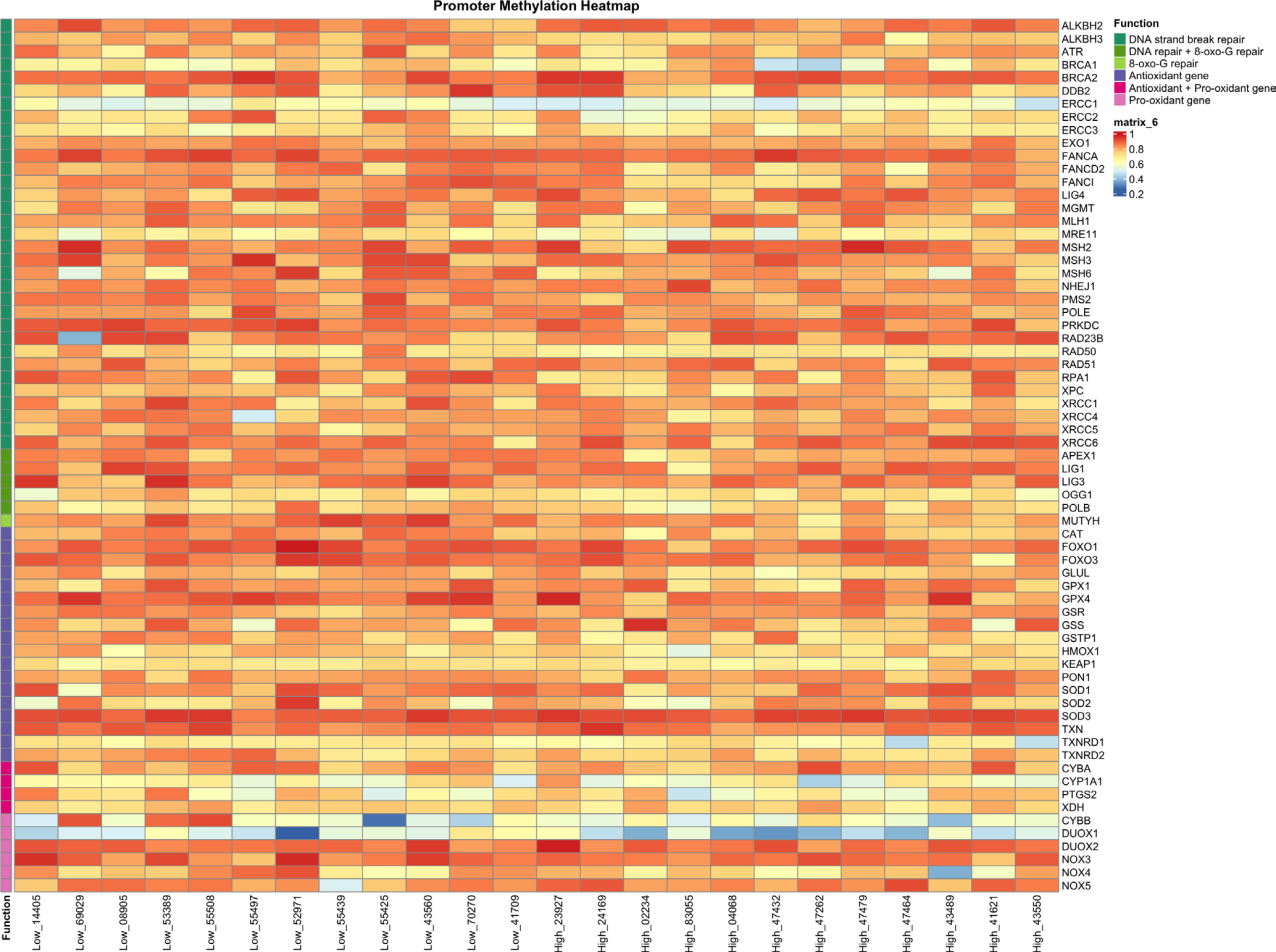
Heat map of promoter methylation for 75 genes relevant to oxidative damage and DNA repair in 24 pet golden retriever dogs. Dogs were in the lowest (Low) and highest (High) quintiles of estimated aggregate systemic exposures to the herbicides 2,4‐D and glyphosate and the volatile organic compounds benzene, xylene and 1,3‐butadiene, out of 60 dogs previously screened [12, 13]. The mean methylation index for each gene (averaged across all CpG sites within each gene) are depicted, with 1 (darkest red) indicating complete methylation and 0 (darkest blue) indicating no methylation. Some genes were assigned to more than one function among DNA strand break repair, 8‐oxoguanine repair, antioxidant and pro‐oxidant activities. Heatmap generated in R using the pheatmap package.

**FIGURE 4:**
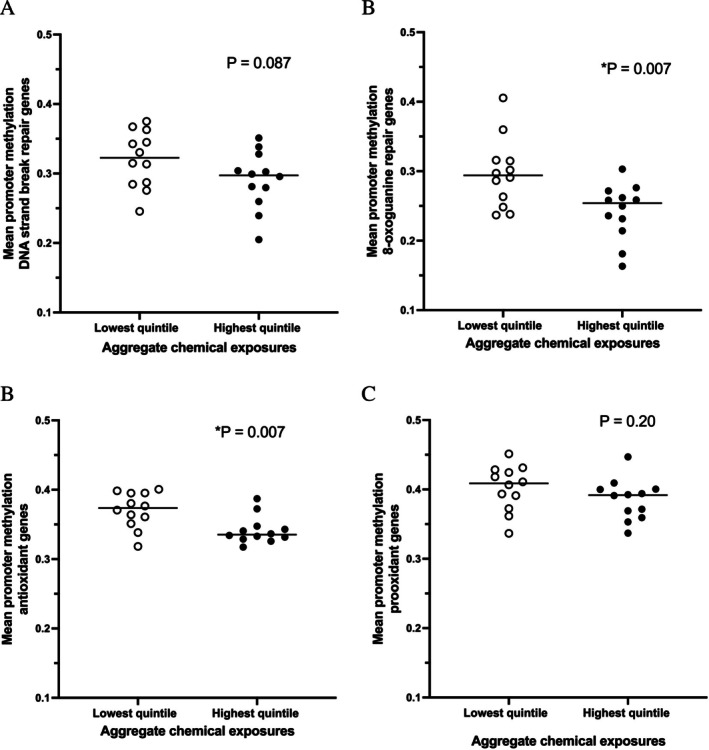
Scatter plots of mean promoter CpG methylation indices for four groups of genes in golden retriever dogs, grouped by the highest and lowest quintiles of estimated aggregate whole blood exposures for the herbicides 2,4‐D and glyphosate and the volatile organic compounds benzene, xylene and 1,3‐butadiene out of 60 dogs surveyed [12, 13]. (A) Insignificant decrease in promoter methylation in DNA repair genes with higher chemical exposures. (B) Signicant decrease in promoter methylation in 8‐oxoguanine genes with higher chemical exposures (*p = 0.007). (C) Significant decrease in promoter methylation in antioxidant genes with higher chemical exposures (*p = 0.007). (D) No difference in average promoter methylation across pro‐oxidant genes between chemical exposure groups. Horizontal lines represent the median within each group; this was chosen for consistency because one dataset (highest quintile antioxidant gene methylation) was not normally distributed.

A heat map for promoter methylation of the 75 genes of interest is shown in Figure 3. Average promoter methylation across DNA repair or pro‐oxidant gene groups was not significantly different between high and low chemical exposure groups (*p* = 0.09 and *p* = 0.20, respectively; Figure 4A,D). Contrary to our initial hypothesis, however, average promoter methylation across antioxidant genes was significantly lower with higher aggregate chemical exposures (median 0.335 for the highest exposure quintile versus 0.374 for the lowest exposure quintile; *p* = 0.007; Figure 4C). This association was still significant when adjusted for age, sex/neuter status and lymphoma diagnosis (*β* = −0.030, *p* = 0.009; Table 2).

**TABLE 2:** Multivariable linear regression of the association between aggregate herbicide and VOC exposures (highest vs. lowest quintiles) and mean promoter methylation of antioxidant genes, adjusting for age, sex/neuter status and lymphoma diagnosis in golden retriever dogs. Effect sizes represent the change in mean promoter methylation attributed to each variable (e.g., high vs. low exposure).

Term	Effect size (*b*)	Standard error	*t* value (*β*/standard error)	*p*	95% CI lower	95% CI upper
Intercept	0.357	0.020	18.128	0.000	0.215	0.371
Binary exposure: high vs. low	−0.030	0.010	−2.901	0.009*	−0.094	−0.011
Age	0.002	0.003	0.872	0.394	−0.007	0.013
Sex neuter group: MN vs. other	0.002	0.010	0.170	0.867	−0.057	0.024
Condition: lymphoma vs. control	0.005	0.011	−0.013	0.990	−0.056	0.032

*Note:* High chemical exposure was still significantly associated with relatively lower promoter methylation of antioxidant genes when these covariates were considered * (*β* = −0.030, *p* = 0.009). No other covariates (age, sex/neuter status or lymphoma status) were significantly associated with antioxidant gene promoter methylation in this model.

Abbreviations: CI, confidence interval; MN, neutered male; *β*, regression coefficient.

Also contrary to our initial hypothesis, average promoter methylation across 8‐oxoguanine genes was significantly lower with higher chemical exposures (median 0.254 for the highest exposure quintile versus 0.2941 for the lowest exposure quintile; *p* = 0.007; Figure 4B). This association remained significant when adjusted for age, sex/neuter status and lymphoma diagnosis (*β* = −0.053, *p* = 0.015; Table 3). Data for individual genes are shown in Tables S1 and S2.

**TABLE 3:** Multivariable linear regression of the association between estimated aggregate herbicide and VOC exposures (high vs. low quintile) and mean promoter methylation of 8‐oxoguanine repair genes, adjusting for age, sex/neuter status and lymphoma diagnosis.

Term	Effect size (*b*)	Standard error	*t* value (*β*/standard error)	*p*	95% CI lower	95% CI higher
Intercept	0.293	0.038	7.818	0.000	0.215	0.371
Binary exposure: high vs. low	−0.053	0.020	−2.666	0.015*	−0.094	−0.011
Age	0.003	0.005	0.597	0.558	−0.007	0.013
Sex neuter group: MN vs. other	−0.016	0.019	−0.830	0.417	−0.057	0.024
Condition: lymphoma vs. control	−0.012	0.021	−0.556	0.584	−0.056	0.032

*Note:* Effect sizes represent the change in mean methylation per category (e.g., high vs. low exposure, or female spayed vs. female intact). High chemical exposure retained a significant association with lower mean promoter methylation of 8‐oxoguanine repair genes in this multivariable model (*β* = −0.053, *p* = 0.015). No other covariates (age, sex/neuter status, or lymphoma status) were significantly associated with 8‐oxoguanine promoter methylation in this analysis.

Abbreviations: CI = confidence interval; *β* = regression coefficient.

* Significant.

### 3.4 Gene Promoter Methylation and DNA Damage

**FIGURE 5:**
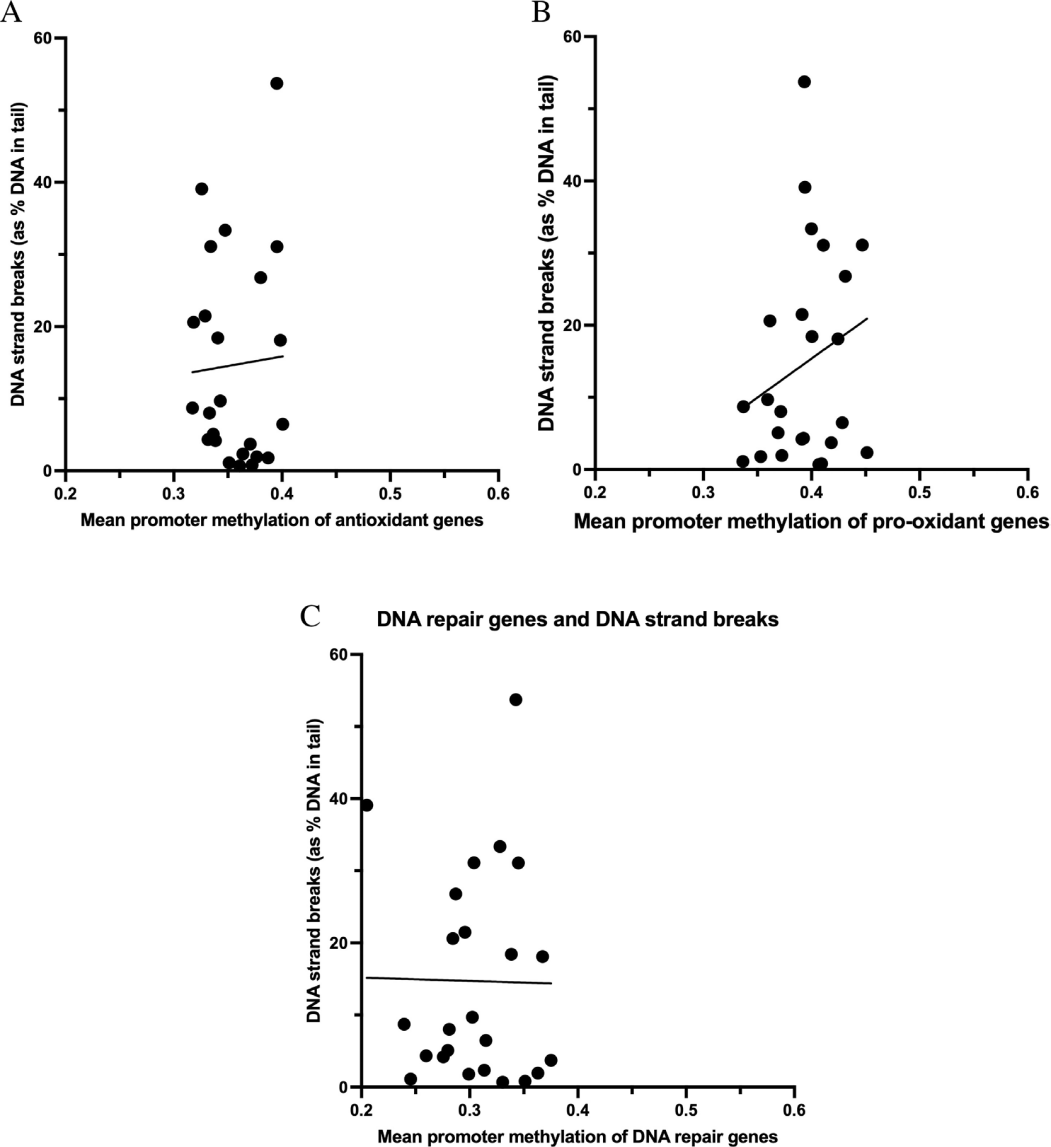
Simple linear regression examining the relationship between DNA strand breaks measured by the comet assay and its relationship with mean promoter methylation of genes in three different pathways. (A) No association between DNA strand breaks and mean promoter methylation of antioxidant genes (*β* = 26.41, *p* = 0.82). (B) No association between DNA strand breaks and mean promoter methylation of pro‐oxidant genes (*β* = 108.0, *p* = 0.27). (C) No association between DNA strand breaks and mean promoter methylation of DNA repair genes (*β* = −4.57, *p* = 0.95). *β* = slope and regression coefficient.

**FIGURE 6:**
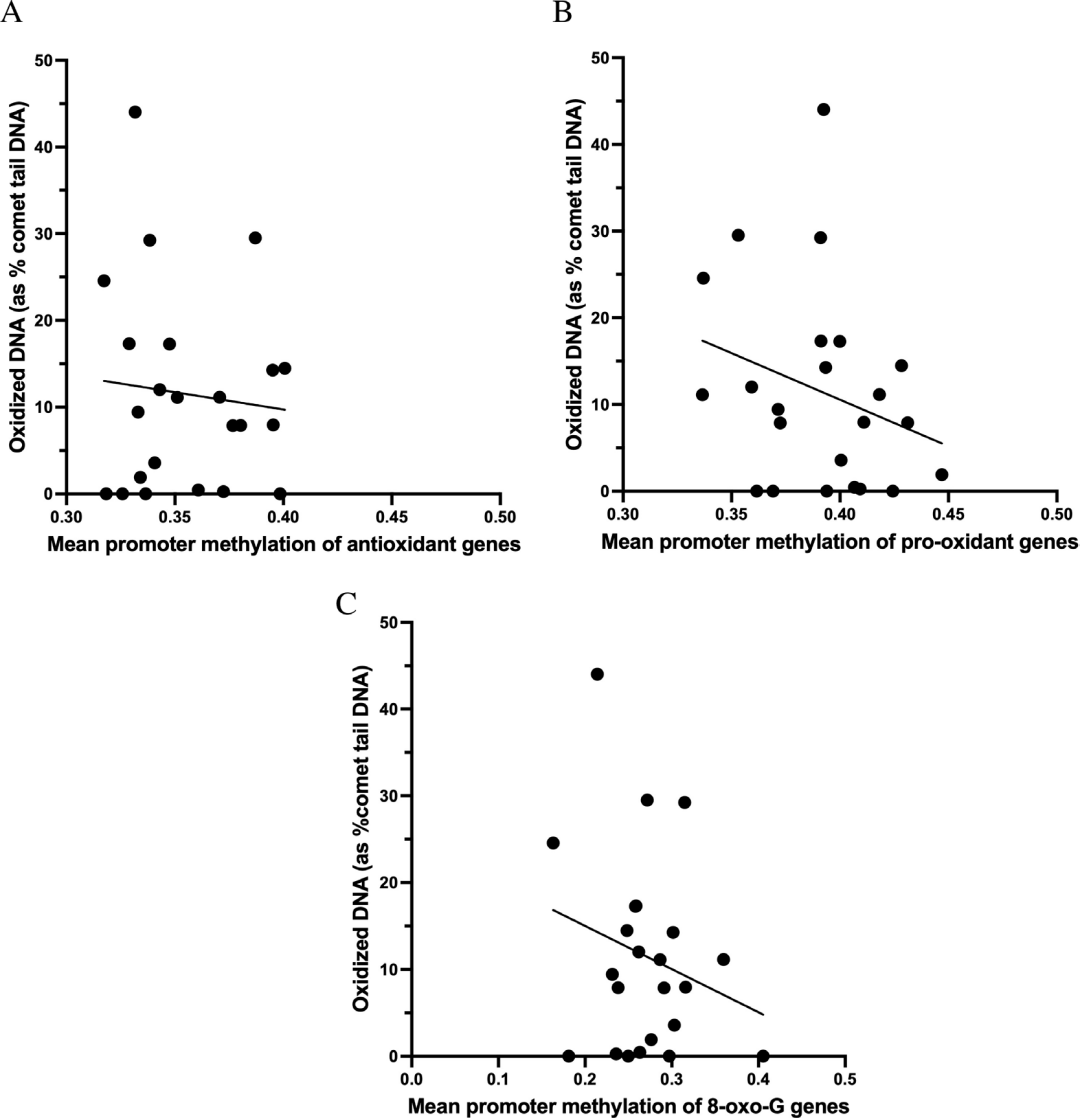
Simple linear regression examining the relationship between oxidized DNA damage measured by the FPG‐modified comet assay, and its relationship with mean promoter methylation of genes in three different pathways. (A) No association between oxidized DNA damage and mean promoter methylation of antioxidant genes (*β* = −39.87, *p* = 0.67) (B) No association between oxidized DNA damage and pro‐oxidant genes (*β* = −107.4, *p* = 0.2). (C) No association between oxidized DNA damage and 8‐oxoguanine repair genes (*β* = −49.74, *p* = 0.3).

No significant associations were observed between DNA strand breaks and mean promoter methylation of antioxidant (*β* = 26.41, *p* = 0.82; Figure 5A), pro‐oxidant (*β* = 108.0, *p* = 0.27; Figure 5B) or DNA repair (*β* = −4.57, *p* = 0.95; Figure 5C) genes. Similarly, no significant associations were observed between oxidised DNA damage and mean promoter methylation of antioxidant (*β* = −39.87, *p* = 0.67; Figure 6A), pro‐oxidant (*β* = −107.4, *p* = 0.20; Figure 6B) or 8‐oxoguanine repair (*β* = −49.74, *p* = 0.30; Figure 6C) gene pathways.

## 4 Discussion

Global DNA methylation levels were consistently high across our study group (78%–82%), with no significant differences observed between chemical exposure groups. Our findings are consistent with similarly high global methylation levels in whole blood reported in three dogs using Oxford Nanopore sequencing (~75%) [29]. In healthy humans, global methylation in peripheral blood also falls within this range (~74%) [30].

Chemical exposures, however, can lead to global hypomethylation in people. Specifically, both low‐level benzene exposure in vivo [31] and glyphosate exposures in vitro [19] are associated with relative global hypomethylation. Global hypomethylation leads to genomic instability [32], and is a possible mechanism for chemically induced DNA damage and cancer risk [31]. In the present study, we did not observe exposure‐related differences in global DNA methylation in our sample of golden retriever dogs. These findings could reflect the use of only a single timepoint, the small sample size, or inaccuracy in reverse dosimetry estimates for chemical exposures in dogs. However, these findings to date do not support the hypothesis that global DNA hypomethylation is a likely mechanism of VOC‐ or herbicide‐associated DNA damage in pet dogs.

Antioxidant and 8‐oxoguanine repair genes in peripheral blood showed lower promoter methylation with higher aggregate chemical exposures, which was inconsistent with our initial hypothesis that chemical stress might suppress antioxidant defenses via promoter hypermethylation. These associations were still significant when considering age, sex/neuter status and lymphoma diagnosis. Relative hypomethylation of antioxidant and 8‐oxoguanine repair genes could represent a response to, rather than a mechanism of, chemically‐associated DNA damage. However, the effect sizes were modest and of unclear clinical biologic significance. Further assessment should include in vitro experiments to directly evaluate the effects of these chemicals on promoter methylation and subsequent expression of antioxidant and 8‐oxoguanine repair genes in canine lymphoid cells in vitro.

Interestingly, we observed no associations between chemical exposures and methylation of pro‐oxidant or DNA repair genes in this group of dogs. We also found no associations between promoter methylation across any of the selected gene groups and measured DNA strand breaks or oxidative DNA residues in whole blood. In contrast, benzene exposure in people is associated with relative hypomethylation of the DNA repair gene *MGMT* as well as DNA damage in whole blood [21]. A larger sample size might allow a more granular assessment of individual gene methylation patterns in association with individual chemical exposures in dogs.

This study has important limitations. First, our sample size was constrained by sequencing costs, which decreased statistical power for multivariable modelling and individual gene methylation assessments. We chose Oxford nanopore sequencing because it could provide a wealth of data for future unbiased analyses and hypothesis testing beyond targeted methylation analyses. However, this approach was also expensive, so we chose the highest and lowest quintiles of aggregate exposures as a best approach to detect chemical exposure‐associated patterns. This approach did not allow us to assess the chemical exposures individually, which could have obscured a specific association with one of the individual VOCs or herbicides.

Whole blood DNA damage and methylation indices were measured at a single time point, which prevented assessment of temporal relationships. Importantly, whole blood DNA is derived from heterogenous subsets of leukocytes, and individual differences in lymphocyte composition could cloud detection of cell‐specific methylation patterns. No dogs had evidence of leukaemic cells noted on their CBCs at T‐1y, or at the time of lymphoma diagnosis for cases. However, specific tests were not performed to check for clonal cells in the circulating blood. If such cells were present at T‐1y but not detected clinically, then the methylation patterns observed at T‐1y could certainly reflect, in part, undetected neoplastic cells. It is also a limitation that the highest and lowest exposure dogs were no longer as closely matched for age and sex (Table 1), although differences in median ages between exposure groups (6.2 versus 5.8 years) are unlikely to be clinically significant.

We chose a promoter window of −1000 to 0 bp from the transcription start site [27]. While this range captures core promoter regions, it excludes the 5′ untranslated region (5′‐UTR), which can also undergo differential methylation [33]. In addition, we did not consider the location of individual methylation sites within target gene promoters, and methylation at specific regulatory elements can have more pronounced epigenetic effects [34].

While our study focused on DNA methylation, environmental chemical exposures can influence gene expression through other epigenetic mechanisms, such as histone modifications that can precede, accompany, or occur independently of DNA methylation changes [35]. Future integrative studies that assess histone modifications using chromatin immunoprecipitation sequencing (ChIP‐seq) or assay for transposase‐accessible chromatin using sequencing (ATAC‐seq) [36], along with methylation profiling, would help identify the range of epigenetic responses in environmentally exposed dogs.

Altogether, these findings support the hypothesis that aggregate environmental herbicide and VOC exposures lead to modest decreases in promoter methylation of antioxidant and 8‐oxoguanine repair pathways in pet dogs. Rather than providing an epigenetic mechanism for chemically‐associated genomic instability, decreased targeted promoter methylation might serve as a compensatory response to chemically‐associated DNA damage in dogs. In vitro experiments are needed to determine whether these exposures directly lead to increased expression of these compensatory pathways in canine lymphoid cells.

## Funding

This work was funded by a Research Forward grant from the Office of the Vice Chancellor for Research at the University of Wisconsin‐Madison. A.N.T. was supported by TL1TR002375 within the Institute for Clinical and Translational Research at the University of Wisconsin‐Madison, supported by U54TR002373, and the University of Wisconsin‐Madison Office of the Vice Chancellor for Research.

## Ethics Statement

Data were provided from the Morris Animal Foundation's Golden Retriever Lifetime Study (GRLS) with deidentified study ID numbers. Dogs were enrolled in GRLS with owner informed consent, obtained by the Morris Animal Foundation. An independent Animal Welfare Advisory Board of Morris Animal Foundation reviewed and approved the study protocol. This research adheres to *Veterinary and Comparative Oncolog*y ethical policies and guidelines for humane animal treatment.

## Conflicts of Interest

The authors declare no conflicts of interest.

## Supporting information


**Figure S1:** Depiction of genome‐wide methylation patterns in peripheral blood DNA from pet golden retrievers grouped in the highest (high; *n* = 12) and lowest (low; *n* = 12) estimated aggregate VOC and herbicide exposures out of 60 dogs.
**Table S1:** Thirty‐eight genes involved in DNA repair and their mean promoter methylation scores across 24 pet golden retrievers.
**Table S2:** Genes involved in 8‐oxoguanine repair (8‐oxo‐G; *n* = 5), antioxidant pathways (*n* = 22) and pro‐oxidant pathways (*n* = 10) and their mean promoter methylation scores across 24 pet golden retrievers.

## Data Availability

The Oxford Nanopore sequencing data that support the findings of this study will be made available through the NCBI sequence read archive (SRA) upon manuscript acceptance https://dataview.ncbi.nlm.nih.gov/object/PRJNA1314263?reviewer=gsifgd038mmjc2o0hqv4u7j0i8.
