# Supplementary material for: Chemical Exposures, DNA Methylation and Whole Blood DNA Damage in Pet Golden Retrievers
Source: Vet Comp Oncol. 2026 Apr 19;24(3):433–43. doi: 10.1111/vco.70066 (PMC13456616; doi:10.1111/vco.70066)
Supplement: Supplementary file 1 [file VCO-24-433-s001.docx]

**Supplemental Table S1**: Thirty-eight genes involved in DNA repair and their mean promoter methylation scores across 24 pet golden retrievers. Dogs were selected by the highest and lowest quintiles of aggregate systemic exposures to environmental chemicals (the herbicides 2,4-D and glyphosate and the VOCs benzene, xylene, and 1,3-butadiene) previously estimated for 60 golden retrievers.^14^

| **DNA repair gene** | **Mean promoter methylation, highest exposure quintile** | **Mean promoter methylation, lowest exposure quintile** |
| --- | --- | --- |
| ALKBH2 | 0.343 | 0.283 |
| ALKBH3 | 0.249 | 0.333 |
| APEX1 | 0.209 | 0.271 |
| ATR | 0.252 | 0.277 |
| BRCA1 | 0.180 | 0.234 |
| BRCA2 | 0.349 | 0.339 |
| DDB2 | 0.643 | 0.698 |
| ERCC1 | 0.179 | 0.209 |
| ERCC2 | 0.200 | 0.251 |
| ERCC3 | 0.285 | 0.279 |
| EXO1 | 0.270 | 0.360 |
| FANCA | 0.477 | 0.537 |
| FANCD2 | 0.252 | 0.270 |
| FANCI | 0.326 | 0.467 |
| LIG1 | 0.309 | 0.361 |
| LIG3 | 0.239 | 0.306 |
| LIG4 | 0.533 | 0.427 |
| MGMT | 0.250 | 0.277 |
| MLH1 | 0.235 | 0.275 |
| MRE11 | 0.289 | 0.360 |
| MSH2 | 0.334 | 0.337 |
| MSH3 | 0.253 | 0.338 |
| MSH6 | 0.225 | 0.302 |
| NHEJ1 | 0.431 | 0.396 |
| OGG1 | 0.225 | 0.263 |
| PMS2 | 0.277 | 0.377 |
| POLB | 0.249 | 0.249 |
| POLE | 0.255 | 0.251 |
| PRKDC | 0.282 | 0.344 |
| RAD23B | 0.317 | 0.261 |
| RAD50 | 0.186 | 0.206 |
| RAD51 | 0.301 | 0.297 |
| RPA1 | 0.183 | 0.247 |
| XPC | 0.219 | 0.235 |
| XRCC1 | 0.264 | 0.333 |
| XRCC4 | 0.235 | 0.292 |
| XRCC5 | 0.416 | 0.352 |
| XRCC6 | 0.311 | 0.281 |

**Supplemental Table S2:** Genes involved in 8-oxoguanine repair (8-oxo-G; n = 5), antioxidant pathways (n = 22) and pro-oxidant pathways (n = 10) and their mean promoter methylation scores across 24 pet golden retrievers. Dogs were selected by the highest and lowest quintiles of aggregate systemic exposures to chemicals (the herbicides 2,4-D and glyphosate and the VOCs benzene, xylene, and 1,3-butadiene) previously estimated for 60 golden retrievers.^14^

| **Gene name** | **Pathway** | **Mean promoter methylation, highest exposure quintile** | **Mean promoter methylation, lowest exposure quintile** |
| --- | --- | --- | --- |
| APEX1 | 8-oxo-G repair | 0.209 | 0.271 |
| CAT | Antioxidant gene | 0.193 | 0.271 |
| CYBA | Antioxidant gene | 0.260 | 0.279 |
| CYBB | Pro-oxidant gene | 0.356 | 0.352 |
| CYP1A1 | Antioxidant gene | 0.164 | 0.191 |
| CYP1A1 | Pro-oxidant gene | 0.164 | 0.191 |
| DUOX1 | Pro-oxidant gene | 0.122 | 0.166 |
| DUOX2 | Pro-oxidant gene | 0.874 | 0.871 |
| FOXO1 | Antioxidant gene | 0.308 | 0.376 |
| FOXO3 | Antioxidant gene | 0.292 | 0.367 |
| GLUL | Antioxidant gene | 0.205 | 0.265 |
| GPX1 | Antioxidant gene | 0.302 | 0.301 |
| GPX4 | Antioxidant gene | 0.292 | 0.351 |
| GSR | Antioxidant gene | 0.746 | 0.743 |
| GSS | Antioxidant gene | 0.367 | 0.350 |
| GSTP1 | Antioxidant gene | 0.280 | 0.318 |
| HMOX1 | Antioxidant gene | 0.224 | 0.270 |
| KEAP1 | Antioxidant gene | 0.221 | 0.228 |
| LIG1 | 8-oxo-G repair | 0.309 | 0.361 |
| MUTYH | 8-oxo-G repair | 0.219 | 0.340 |
| NOX3 | Pro-oxidant gene | 0.856 | 0.866 |
| NOX4 | Pro-oxidant gene | 0.164 | 0.240 |
| NOX5 | Pro-oxidant gene | 0.289 | 0.278 |
| OGG1 | 8-oxo-G repair | 0.225 | 0.263 |
| POLB | 8-oxo-G repair | 0.249 | 0.249 |
| PON1 | Antioxidant gene | 0.756 | 0.764 |
| PTGS2 | Antioxidant gene | 0.155 | 0.184 |
| PTGS2 | Pro-oxidant gene | 0.155 | 0.184 |
| SOD1 | Antioxidant gene | 0.268 | 0.282 |
| SOD2 | Antioxidant gene | 0.208 | 0.225 |
| SOD3 | Antioxidant gene | 0.913 | 0.900 |
| TXN | Antioxidant gene | 0.362 | 0.379 |
| TXNRD1 | Antioxidant gene | 0.241 | 0.299 |
| TXNRD2 | Antioxidant gene | 0.243 | 0.298 |
| XDH | Antioxidant gene | 0.614 | 0.589 |
| XDH | Pro-oxidant gene | 0.614 | 0.589 |

**Supplemental Figure S1:** Depiction of genome-wide methylation patterns in peripheral blood DNA from pet golden retrievers grouped in the highest (High; n = 12) and lowest (Low; n =12) estimated aggregate VOC and herbicide exposures out of 60 dogs.^12,13^ Genome-wide DNA methylation patterns were visualized using the **karyoploteR** package in R. DNA methylation data summarized at 100 kb resolution were first converted into GRanges objects containing genomic coordinates and mean methylation frequencies for each bin. The figure was generated using chromosome length information from the canine reference genome assembly CanFam6.0, with only primary autosomes and the X chromosome included. For each chromosome, the mean percent methylation across the genome for each group of dogs was displayed as a heatmap track, where yellow indicates high methylation levels (values approaching 1) and purple indicates low methylation levels (values approaching 0). CpG islands are plotted immediately adjacent to each chromosome as a separate annotation track. Genomic regions corresponding to the 67 targeted genes relevant to DNA repair and redox status are overlaid on the chromosomes as red rectangles. Gene positions and lengths were obtained from NCBI RefSeq genome annotations for CanFam6, and gene symbols were annotated below the corresponding regions. The figure is best viewed with a zoom-in function.
